# Breast MRI in the era of diffusion weighted imaging: do we still need signal-intensity time curves?

**DOI:** 10.1007/s00330-019-06346-x

**Published:** 2019-07-29

**Authors:** Matthias Dietzel, Stephan Ellmann, Rüdiger Schulz-Wendtland, Paola Clauser, Evelyn Wenkel, Michael Uder, Pascal A. T. Baltzer

**Affiliations:** 1 Department of Radiology, University Hospital Erlangen, Maximiliansplatz 1, 91054 Erlangen, Germany; 2 Department of Biomedical Imaging and Image-Guided Therapy, Division of Molecular and Gender Imaging, Medical University of Vienna, Waehringer-Guertel 18-20, 1090 Vienna, Austria

**Keywords:** Magnetic resonance imaging, Breast neoplasms, Differential diagnosis, Diffusion magnetic resonance imaging, Diagnostic techniques and procedures

## Abstract

**Objective:**

Dynamic contrast-enhanced imaging of the initial (IP) and delayed phase (DP) is an integral part of any clinical breast MRI protocol. Furthermore, DWI is increasingly used as an *add-on* sequence by the breast-imaging community. We investigated whether DWI could be used as a *substitute* DP.

**Material and methods:**

One hundred thirty-two consecutive patients with equivocal or suspicious findings at ultrasound and/or mammography received a full diagnostic breast MRI according to international recommendations. Histopathological verification served as reference standard. We evaluated three sections of the MRI protocol: IP, DP, and apparent diffusion coefficient (ADC) maps derived from DWI. Circular ROIs (regions of interest, mean size 5–10 mm^2^) were drawn into the enhancing parts of the lesion (first postcontrast). ROIs were transferred to the corresponding location on ADC maps and IP and DP images. Mean ROI values were investigated signal intensity (SI): (1) Initial-phase enhancement = (SI(IP) − SI(precontrast))/SI(precontrast); (2) Delayed-phase enhancement = (SI(DP) − SI(IP))/SI(IP); (3) ADC. Multiparametric combinations were computed using logistic regression analysis: (1) IP+: Initial-phase enhancement and ADC; (2) Curve: Initial-phase enhancement and delayed-phase enhancement; (3) Curve+: Curve and ADC. The diagnostic performances of these feature combinations to diagnose malignancy were compared by the area under the receiver-operating characteristics curve (AUC).

**Results:**

One hundred thirty-two patients (age: mean = 57.1 years, range 23–83 years) with 145 lesions were included (malignant/benign 101/44). IP+ (AUC = 0.877) outperformed Curve (AUC = 0.788, *p* = 0.03). Curve+ was not superior to IP+ (*p* = 1).

**Conclusion:**

DWI could substitute DP. Because DWI is typically used as an add-on to IP and DP, our results might help to abbreviate and to simplify current practice of breast MRI.

**Key Points:**

*• DWI provides similar but superior diagnostic information for diagnosis of malignancy in enhancing breast lesions compared to DP.*

*• Adding DP to DWI does not provide incremental information to distinguish benign from malignant lesions.*

*• DWI could substitute DP. As DWI is typically used as an add-on to IP and DP, our findings might help to abbreviate and to simplify current breast MRI practice.*

## Introduction

Breast magnetic resonance imaging (MRI) provides the highest sensitivity and the highest negative predictive value in the radiological diagnosis of breast cancer [1, 2]. The current approach to breast MRI is multiparametric. Therefore, assessment of MRI requires evaluation of multiple parameters derived from numerous sequences [3–5]. Accordingly, interpretation of MRI is complex and requires special training. This is one reason for the slow adoption of this technique by its stakeholders [6].

Interpretation of MRI is based on lesion *identification* and lesion *classification*. The identification of a lesion is generally based on the enhancement during the initial phase (IP) scanned 1–2 min after injection of contrast agent [4, 5]. Once a lesion is identified, it must be classified as being benign or malignant. For this purpose, morphologic criteria are applied during the IP or on T2-weighted images [4, 5]. As morphologic criteria can feature overlapping characteristics in benign and malignant lesions, the dynamic enhancement within the delayed phase (DP) has to be assessed as well; hereby, the change of the signal intensity between the IP and the last scan after contrast agent injection is investigated by semiquantitative metrics. As the DP provides essential diagnostic information, it is considered an integral part of every standard breast MRI protocol [4, 5, 7].

Besides morphologic and dynamic criteria, numerous additional MRI techniques have been investigated [8–14]. In this context, diffusion-weighted imaging (DWI) is arguably the most promising method. Current breast DWI sequences are fast and can provide quantitative imaging data within 2–3 min scanning time. Calculated from raw DWI data, the apparent diffusion coefficient (ADC) is most commonly applied for diagnostic purposes [15, 16]. Nowadays, there is a substantial body of evidence proving that DWI can distinguish benign from malignant enhancing breast lesions [8–14, 16]. As a consequence, DWI is increasingly adopted by the community. A recent survey reports that already 60% of radiologists performing breast MRI use DWI in addition to IP and DP in every examination. Nonetheless, adding DWI to a standard protocol increases scanning time and the level of complexity [17].

DWI is typically used as an add-on to IP and DP in clinical practice. However, both DP curve type and the ADC are influenced by extracellular space properties. It can thus be assumed that the physiological and diagnostic information extracted by these markers may overlap. We therefore hypothesized that DWI could be used as a substitute of DP. If verified, this might abbreviate and simplify current practice of breast MRI.

## Material and methods

### Patients

Informed consent for this retrospective monocentric cross-sectional study was waived by the institutional ethical committee. Diagnostic workup, treatment, and follow-up of patients with suspected or proven breast cancer were performed in accordance with evidence-based guidelines [18]. Data were collected at an academic tertiary care institution and stored in a central database. The breast center consists of the Departments of Gynecology, Oncology, Radiology, Pathology, and Radiation Oncology.

Breast MRI indications were as follows:

1. *Problem solving* of equivocal findings. Such findings received BI-RADS 0, 3, 4a, or 4b assessment in mammography and ultrasound.
2. *Preoperative staging* of findings featuring unambiguous patterns of malignancy in mammography and ultrasound (BI-RADS 4c and 5).

BI-RADS VI cases were not considered, as artifacts due to biopsy could bias the assessment of DWI (bleeding, T2* artifacts, etc.) [14]. The term “BI-RADS” refers to the assessment categories based on mammography and ultrasound.

#### Database

Consecutive patients having received breast MRI over a period of 10 consecutive months following the aforementioned indications were eligible, yielding 950 patients in total.

#### Dataset

By applying the following exclusion criteria to the database, the dataset of the present study was created:

1. Absence of the standard of reference (719 patients)
2. Artifacts (11 patients) or technical failures (insufficient fat saturation, 23 patients)
3. Status after neoadjuvant chemotherapy (39 patients)
4. Absence of a lesion at breast MRI (26 patients)

**Fig. 1:**
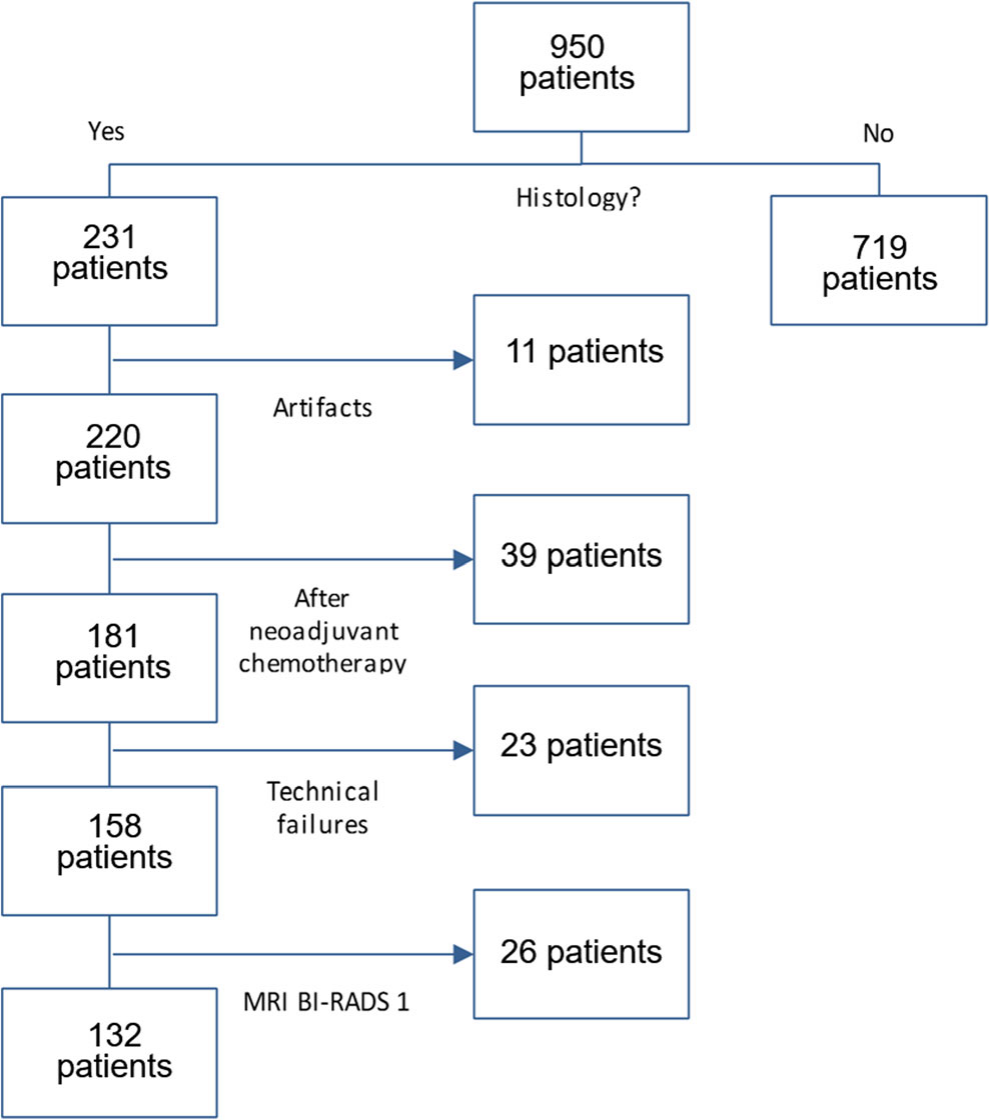
Flowchart demonstrating patient selection toward the final study collective. Note: MRI BI-RADS 1 cases were biopsied due to conventional imaging findings without MRI correlate

A flowchart demonstrating patient selection toward the dataset of the present study is given in Fig. 1. Accordingly, 132 patients were included.

### Standard of reference

Histopathological verification served as the standard of reference (SOR). All histopathologic examinations were performed by a board-certified breast pathologist. Tissue sampling was done in accordance with evidence-based national S3 guidelines [18].

- If the lesion could be visualized by ultrasound, 14-gauge core biopsy was performed under sonographic guidance.
- If the lesion could not be visualized by ultrasound, but in mammography, 9-gauge vacuum-assisted biopsy using stereotactic guidance was executed.
- MRI-guided 9-gauge vacuum-assisted biopsy was reserved for MRI-only lesions.
- In case of discrepant findings between imaging and histology and in lesions with uncertain malignant potential—such as papillomas—surgical excision was performed.

### MRI

One 1.5-T whole-body MRI scanner was used in combination with the vendor-supplied receive-only 4-channel circularly polarized breast array coil (Magnetom Avanto; Siemens Healthineers). A standardized protocol optimized to achieve homogeneous image quality following international recommendations was acquired [3, 5, 19].

Three sections of the protocol were investigated:

1. IP (2:40 min): The precontrast and the first postcontrast scans were considered as IP [4, 5].
2. DP (+ 5 min): Five additional postcontrast scans were acquired [3, 5, 19].
3. DWI (+ 2:30 min) [14, 15, 20, 21].

**Fig. 2:**
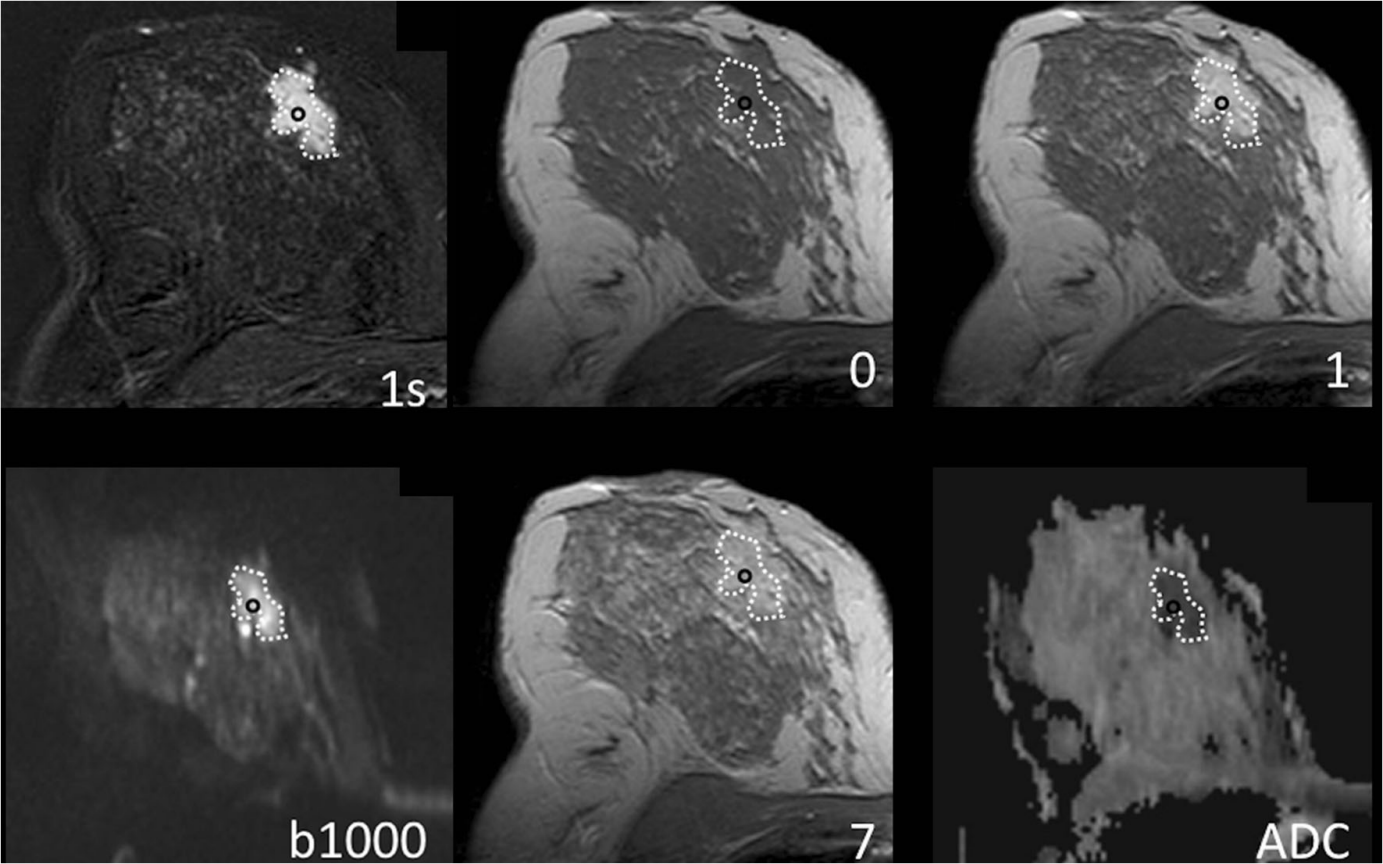
Breast MRI of a 41-year-old woman with invasive ductal cancer grade 2. Numbers denote acquisition time points of T1-weighted dynamic gradient echo images after intravenous contrast medium injection in minutes (**0** = precontrast acquisition). **1s** equals first subtraction. The dotted line delineates the lesion from the surrounding breast parenchyma. The lesion is depicted as an ill-defined mass lesion with fast initial-phase enhancement and wash-out during the delayed phase. The region of interest to extract the diagnostic information used in this work is highlighted by a black circle

The examination started with a bilateral axial echo planar imaging (EPI) DWI sequence (GRAPPA factor 2, TR 3500 ms, TE_eff_ 73 ms, echo distance 0.95 ms, 6 averages, 3 *b*-values—0, 750, and 1000 s/mm^2^, diffusion mode: 3-Scan Trace, spectral fat saturation, in plane resolution 1.8 × 1.8 mm^2^, slice thickness 6 mm, matrix 192 × 192 pixels, FOV 350 × 350 mm, acquisition time 2:30 min). ADC maps were calculated from raw diffusion-weighted images using all *b*-values and applying the standard monoexponential regression approach performed by the scanner software automatically. The b0 noise level was set to ≥ 30 arbitrary units [22]. A clinical example is shown in Fig. 2.

The DWI was followed by the initial and delayed phase (IP, DP). For both, a dynamic T1-weighted radiofrequency spoiled gradient echo sequence was used (FLASH 2D, GRAPPA factor 2, TR 113 ms, TE 5 ms, flip angle 80°, temporal resolution 1 min). Likewise, the spatial resolution was in accordance with international guidelines (matrix 384 × 384 pixels, FOV 340 × 30 mm) [3]. The dynamic sequence was acquired before and after automated intravenous bolus injection (Spectris, Medrad) of gadopentetate dimeglumine (Magnevist, Bayer Vital) at a dosage of 0.1 mmol/kg body weight followed by 20 ml saline solution. A delay of 30 s after contrast medium application was set prior to the acquisition of postcontrast images under identical tuning conditions for a total of 7 measurements. Considering an average injection time of 10 s and a subsequent delay of 30 s, this gave an acquisition time of 2:40 min for IP and 5:00 min for DP.

### Interpretation of MRI

Examinations were prospectively evaluated by a reader highly experienced in breast MRI (> 500 exams/year) and blinded to the SOR. The reader had access to all relevant clinical data and previous imaging of the patient. The reader was supported by an assistant with special training in DWI and breast MRI (200 breast MRI). The latter provided support in data loading and extraction but did not perform self-reliant data analysis.

Imaging data were analyzed on a dedicated workstation (Multi-Modality Work-Place, Siemens Healthineers). A circular region of interest (ROI) (mean size 5–10 mm^2^) was drawn around the most suspicious enhancing part of the lesion upon the first postcontrast scan. Lesion size was defined as the largest diameter of the enhancing lesion in the IP including perifocal nonmass. ROIs were automatically transferred to the other imaging series, and in case of misalignment due to geometric distortion of DWI, a manual correction was performed. This approach has been investigated previously [15]. Based on mean ROI values, semiquantitative (IP, DP) and quantitative (DWI) parameters were calculated to investigate each section of the protocol:

- IP: Initial-phase enhancement = $\frac{\left({\mathrm{SI}}_{1\mathrm{st}\kern0.17em \mathrm{postcontrast}}-{\mathrm{SI}}_{\mathrm{precontrast}}\right)}{{\mathrm{SI}}_{\mathrm{precontrast}}}$
- DP: Delayed-phase enhancement = $\frac{\left({\mathrm{SI}}_{\mathrm{last}\kern0.17em \mathrm{postcontrast}}-{\mathrm{SI}}_{1\mathrm{st}\kern0.17em \mathrm{postcontrast}}\right)}{{\mathrm{SI}}_{1\mathrm{st}\kern0.17em \mathrm{postcontrast}}}$
- DWI: ADC given as 10^–3^ mm^2^/s

Hereby, SI refers to signal intensity. Parameters were taken from BI-RADS MRI (IP, DP) or from the DWI literature [5, 14, 15, 20, 23].

### Data analysis

Assessment of the SOR by MRI was executed on a “lesion level analysis”, corresponding to a type 5 study by Obuchowski et al [24].

Correlation between single parameters (ADC, initial- and delayed-phase enhancement) was investigated using the Spearman rank correlation coefficient. Direction of correlation was either “positive” (rho > 0) or “negative” (rho < 0). Absolute values of rho were interpreted as “high” (0.70 to 1), “moderate” (0.50 to 0.70), and “low” (0.30 to 0.50) [25, 26].

**Fig. 3:**
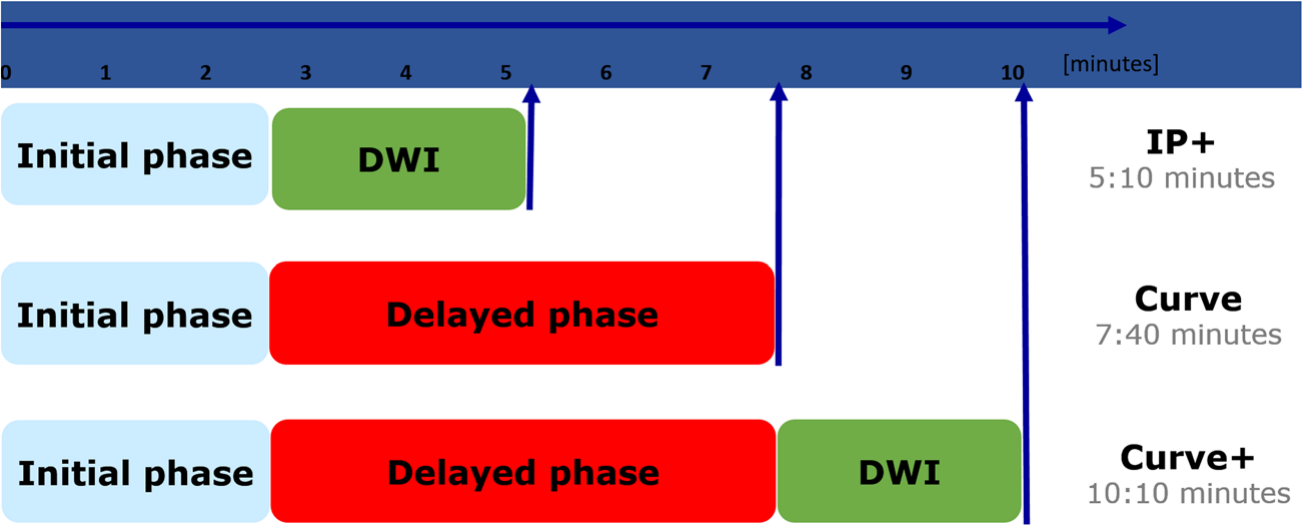
Graphical summary of the three protocols investigated in the present study. The combination of the initial phase (IP) and DWI gave IP+. Requiring a scanning time of 5:10 min, IP+ enables assessment of the initial phase, lesion morphology, and the ADC. The combination of the initial phase (IP) and the delayed phase gave Curve. Requiring a scanning time of 7:40 min, Curve enables assessment of the initial and the delayed phase (washout, plateau, and persistent increase). Integration of DWI into Curve gave Curve+ (scanning time 10:10 min). A potential alternative to be investigated in the future would be interleaved curve: It adds one scan of the DP to the IP+ (scanning time 6:10 min) and hereby combines the potential of Curve+ within a much shorter examination time

We investigated whether DP could be substituted by DWI. For this purpose, three different multiparametric combinations parameter combinations were defined (Fig. 3):

I. IP+: Initial-phase enhancement and ADC
II. Curve: Initial-phase enhancement and delayed-phase enhancement
III. Curve+: Curve and ADC

The diagnostic performance of each protocol was assessed. Hereby, logistic regression with backward feature selection was applied (enter and remove: *p* < 0.05/> 0.1; covariates: ADC, initial/delayed-phase enhancement). This approach allowed to estimate which parameter significantly and independently distinguished benign from malignant lesions. Predictive values were saved for the final analysis.

### Diagnostic accuracy

The area under the receiver-operating characteristics curve (AUC) was calculated and compared as a measure of diagnostic accuracy with lesion type (benign vs. malignant) as the target variable.

The AUC was calculated for single parameters (DWI, IP, and DP) and for parameter combinations (IP+, Curve, Curve+). Either the mean ROI values (single parameters) or the predictive values of the logistic regression (IP+, Curve, Curve+) were used as variables.

Pairwise comparison of corresponding AUC was achieved according to the method described by DeLong et al [27]. All *p* values < 0.05 were considered statistically significant [26].

## Results

### Patients

A flowchart demonstrating patient selection toward the dataset of the present study is given in Fig. 1. Accordingly, 132 patients were included (mean age 57.1 years, range 23–83 years). In these patients, 145 lesions received histological verification and were included into the study. One hundred one lesions were malignant (69.7%; mean age 59.9 years, range 25–83 years) and 44 benign were benign (30.3%; mean age 50.4 years, range 23–74 years).

Diagnoses of benign lesions included fibrocystic changes (*n* = 14; 31.8%), fibroadenoma (*n* = 12; 27.3%), papilloma (*n* = 8; 18.2%), and other nonmalignant findings (*n* = 10; 22.7%). Malignant lesion subtypes consisted of invasive ductal (*n* = 66; 65.3%), invasive lobular (*n* = 8; 7.9%), mixed invasive ductal and lobular (*n* = 11; 10.9%), other invasive cancers (*n* = 6; 5.9%), and ductal carcinoma in situ (*n* = 10; 9.9%). Mean size of breast cancer (20 mm, range 3–80 mm) was larger compared to benign lesions (11.5 mm, range 3–50 mm; *p* < 0.001).

### Single parameters

All single parameters showed significant potential to distinguish benign from malignant lesions (*p* < 0.001). Initial-phase enhancement reached an AUC of 0.743 (standard error (SE) 0.05). The AUC of ADC (AUC = 0.863, SE 0.04) was significantly higher compared to initial-phase enhancement (*p* = 0.02). The diagnostic performance of delayed-phase enhancement reached an AUC of 0.813 (SE 0.04). This value was in between ADC (*p*_DP vs. ADC_ = 0.11) and initial-phase enhancement (*p*_DP vs. IP_ = 0.18).

### Correlation analysis

**Fig. 4:**
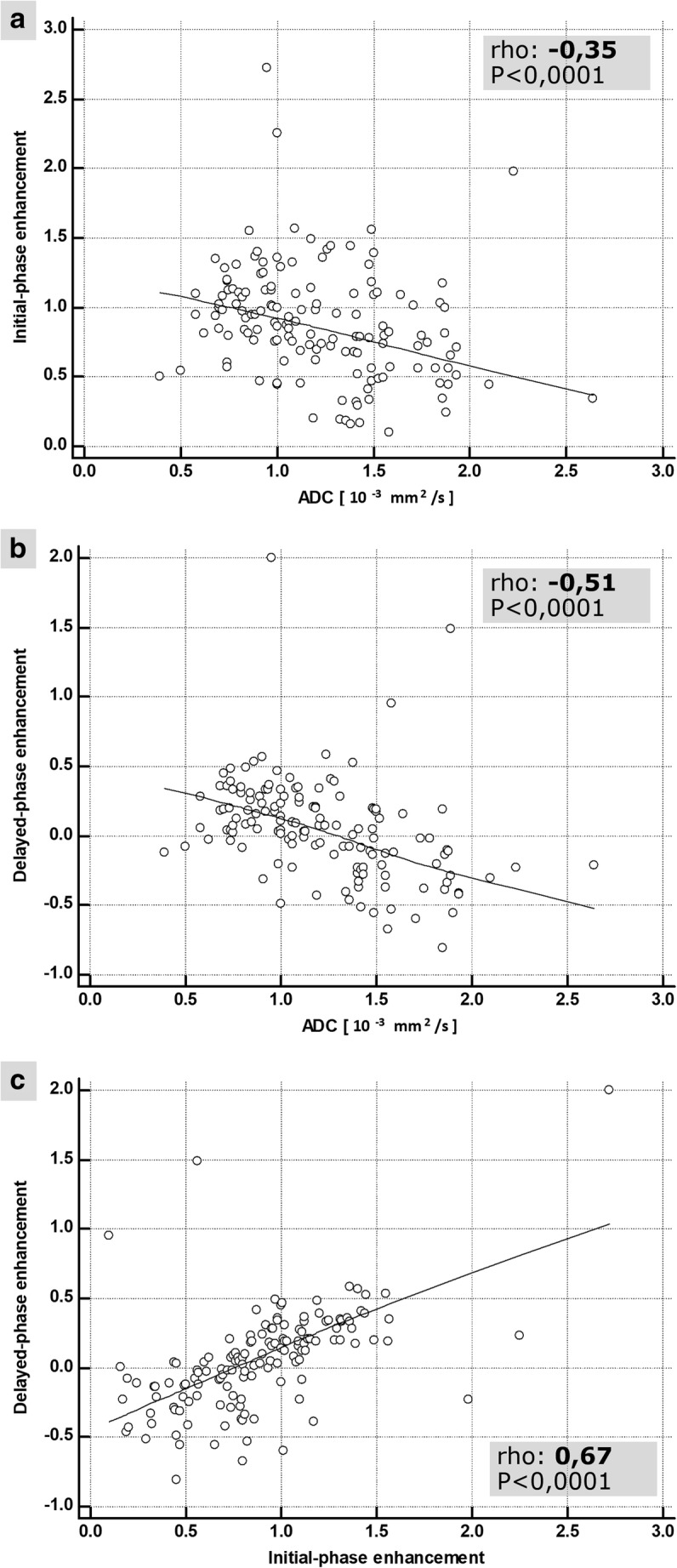
Correlation of the diagnostic information provided by IP, DP, and DWI. The lowest correlation was observed between initial-phase enhancement and the ADC (**a** rho = − 0.35). This is in line with the incremental diagnostic information, if both parameters are used in combination (IP+). In contrast, delayed-phase enhancement showed a stronger correlation both with ADC (**b** rho = − 0.51) and initial-phase enhancement (**c** rho = 0.67). As illustrated in Fig. 5, these correlations could be explained by overlapping pathophysiology: similar to IP, also the DP investigates the vasculature. Comparable to DWI, DP reflects the EES as well

As displayed in Fig. 4, correlation analysis demonstrated different degrees of correlation between the single parameters: There was high correlation between delayed-phase versus initial-phase enhancement (rho = 0.67, 95% confidence interval (CI) = 0.57 to 0.75, *p* < 0.001) and moderate correlation between delayed-phase enhancement versus ADC (rho = − 0.51, CI = − 0.62 to − 0.38, *p* < 0.001). There was low correlation between initial-phase enhancement versus ADC (rho = − 0.35, CI = − 0.49 to − 0.20, *p* < 0.001).

### Multiparametric combinations

Compared to initial-phase enhancement alone, the combined assessment of initial-phase enhancement and ADC (IP+) raised the accuracy by 13.4% (*p* = 0.002; Table 1). The corresponding model of IP+ reached AUC = 0.877 (SE 0.03) and is shown in Table 2. The multiparametric assessment of initial-phase and delayed-phase enhancement (Curve: AUC = 0.788, SE = 0.05) raised the accuracy of IP to a lesser degree (Curve vs. IP+: *p* = 0.03). IP+ could not be further optimized by including DP into the protocol (Curve+ = IP+; *p* = 1).

**Table 1:** Performance of dynamic breast MRI with and without DWI

Protocol	AUC	SE	95% CI
IP	0.743	0.0476	0.664 to 0.812
Curve (IP+DP)	0.788	0.0451	0.712 to 0.851
IP+ (IP+DWI)	0.877	0.0304	0.813 to 0.926
Curve+ (IP+DWI+DP)	Equals IP+		

The initial phase (IP) was evaluated by the initial-phase enhancement. The delayed phase (DP) was evaluated by the delayed-phase enhancement. Multiparametric assessment of IP and DP (Curve) slightly increased the diagnostic performance compared to IP alone (*p* = 0.27). On the other hand, multiparametric assessment of IP and ADC (IP+) significantly increased the performance compared to IP alone by 13.4% (*p* = 0.002). Multiparametric assessment of IP+ and DP (Curve+) yielded identical diagnostic results compared to IP+ (*p* = 1) but required an additional 5 min of scanning time

*AUC* area under the ROC curve, *SE* standard error, *CI* confidence interval

**Table 2:** Logistic regression model for IP+ and Curve+

Variable	Coefficient	SE	*p*	Odds ratio	95% CI
ADC	− 0.035	0.007	< 0.0001	0.966	0.953 to 0.979
Initial enhancement	1.618	0.636	0.01	5.041	1.45 to 17.525
Constant	4.057	1.106	0.0002		

IP+: multiparametric assessment of the initial phase and the ADC. Curve+: multiparametric assessment of IP+ and the delayed phase. As the latter did not contribute to diagnostic accuracy, this model is the same for IP+ and Curve+. For further details, see Table 1

## Discussion

DWI provided similar but superior diagnostic information for diagnosis of malignancy in enhancing breast lesions compared to DP. Adding DP to DWI did not provide incremental information to distinguish benign from malignant lesions. In conclusion, DWI could substitute DP. As DWI is typically used as an add-on to IP and DP, our findings have a potential clinical impact. They provide a rationale to shorten and to simplify current breast MRI practice without losing diagnostic information.

A recent survey from the European Society of Breast Imaging (EUSOBI) gives an overview of current clinical breast MRI practice [17]: Only a minority of radiologists (25%) do not use DWI at all, whereas 60% apply diffusion-weighted imaging in every case. Accordingly, DWI is already widely adopted by the breast MRI community; however, radiologists acquire DWI typically in addition to IP and DP [17]. So DWI is typically used as an add-on to a standard breast MRI protocol in current clinical practice. According to our results, DP might be omitted from this protocol without losing relevant diagnostic information. Therefore, considering the current practice of performing breast MRI, our finding has the potential to shorten examination time.

In its fourth decade of clinical evaluation, breast MRI has still not been fully adopted by its stakeholders. According to Rogers, one critical success factors of any innovation is its complexity [6]. Compared to other innovations in breast imaging, MRI is a technically challenging method. This is an inherent disadvantage of MRI and has been one reason for its slow adoption rate until today [6]. By deleting the DP, our findings provide a rationale to simplify current breast MRI protocols [17]. Following established models to predict success of an innovation, our findings could therefore be helpful to increase adoption rate of breast MRI in general [6].

**Fig. 5:**
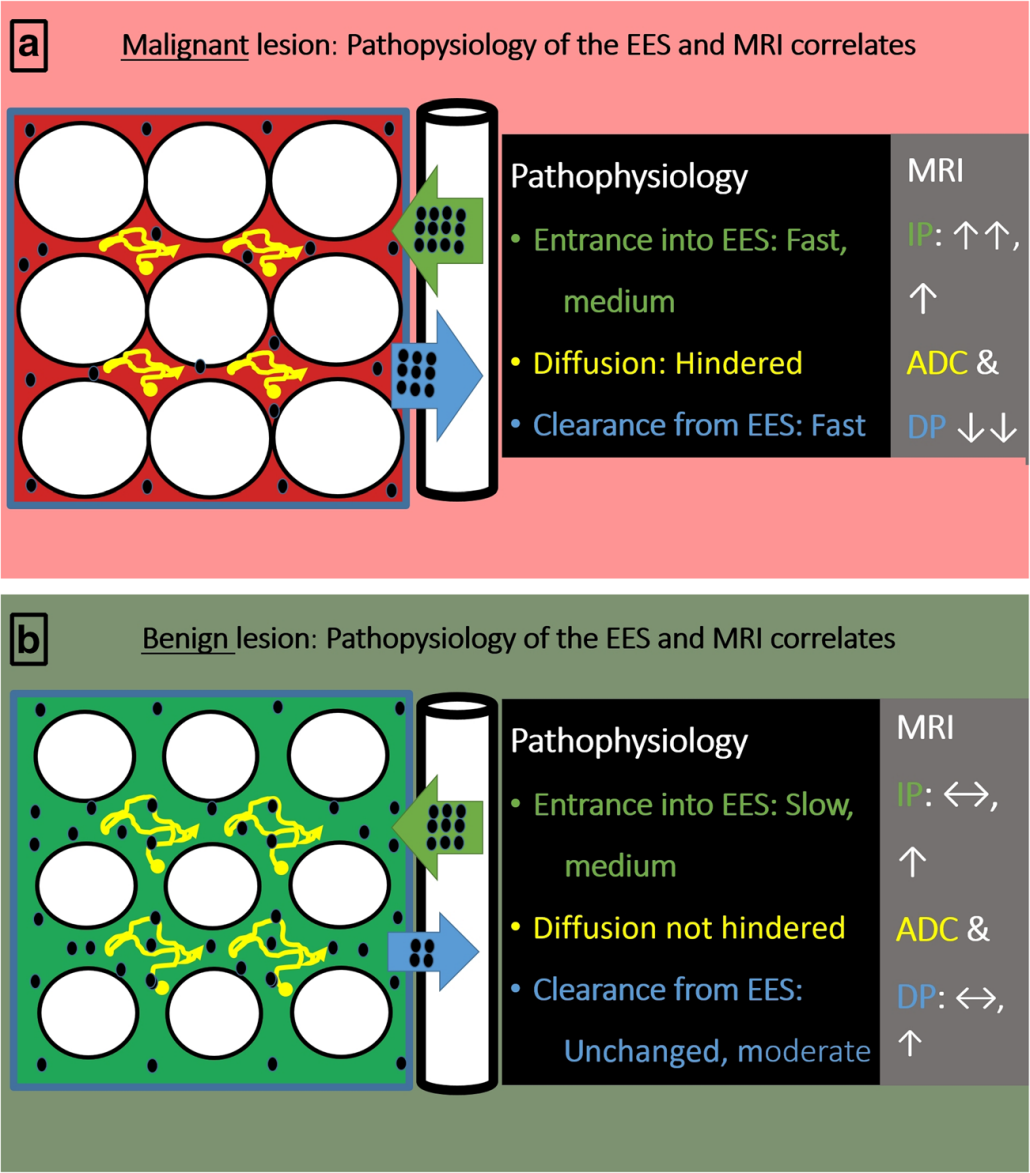
The extravascular extracellular space (EES) could serve as an explanation for the partially overlapping diagnostic information of DWI and DP: **a** in malignant lesions, the vasculature is characterized by an increased permeability. This accelerates the transfer of the contrast agent into the EES and is reflected by a fast initial-phase enhancement rate (IP↑↑**:** fat green arrow with many black dots). Increased permeability also leads to a faster clearance of the contrast agent out of the EES back into the leaky vasculature EES. As a consequence, less contrast agent is present at the end of the DP (fewer black dots). This finding corresponds to a fast washout (DP↓↓: fat blue arrow, many black dots). Of note, the same processes within the EES will also cause an impaired diffusion within the lesion (ADC**↓↓**). **b** Likewise in benign lesions, a high permeability of the vessel will cause a rather fast entrance of the contrast agent into the EES (IP**↑:** fat green arrow, with many black dots). Accordingly, overlapping patterns between benign and malignant lesions can be observed during the IP. In the benign scenario, desmoplastic reactions are less typical. Cellularity of noncancerous lesions may be unchanged compared to physiological conditions or even be decreased. Correspondingly, the interstitial fluid pressure within the EES is typically not increased. As a consequence, a larger volume of contrast agent is retained at the end of the DP (more black dots compared to **a**). This might explain why clearance of the contrast agent out of the EES is slower (typically DP: ↔, ↑: enlarged blue arrow, fewer black dots compared to **a**). Again these pathophysiological considerations are also reflected by the ADC: values are typically increased or normal in benign lesions (typically ADC: ↔, ↑)**.** If T2 black-out artifacts due to fibrotic component are absent, ADC is typically not reduced

As we did not perform a pathological–radiological correlation, we are not able to prove the pathophysiological basis of our findings. Nevertheless, there are some general considerations potentially explaining our key observations (see also Fig. 5). Due to cancer-caused tissue alterations such as increased cellularity, desmoplastic reaction, and increased interstitial fluid pressure, the contrast agent is cleared more quickly from the extravascular extracellular space (EES), causing a fast signal decrease during the DP [8–11, 28]. Similar processes within the EES result in a hindered diffusion corresponding to lower ADC values [8–11, 14, 29]. As both DP and DWI investigate the EES, the correlation between delayed-phase enhancement and the ADC is consistent (Fig. 4).

However, this correlation was only moderate, hinting on further underlying processes. The most important factor to be considered is the architecture of the vessels with a major impact both on the IP and the DP, but not so much on the DWI [8–11, 28]. Accordingly, the low correlation between ADC and the IP is conclusive (rho = − 0.35) [30, 31]. On the other hand, vessel structure significantly contributes both to the delayed- and the initial-phase enhancement [28]. This explains the “high” correlation between IP and DP, resulting in a minor increase of accuracy (4.5%), if IP and DP were combined (“Curve”). In fact, “Curve” even performed marginally (2.5%) worse than DP alone in distinguishing benign from malignant lesions. These findings hint to redundant diagnostic information between IP and DP.

Limitations of our study have to be addressed. There are numerous technical challenges of DWI. Particularly, EPI sequences are prone to artifacts such as ghosting, chemical shift, and distortions—especially at 3 T [14]. All these effects are particularly challenging for breast MRI due to off-center imaging, air–tissue interfaces, and significant fat content in the breasts. Similarly, the in-plane resolution of breast DWI is still not perfect, limiting the assessment of small lesions and subtle changes. However, the fast acquisition time makes DWI less susceptible to motion artifacts. The latter can be challenging in the interpretation of the DP [32]. Finally, the quantitative nature of the ADC is not without controversy, and there is ongoing research how to achieve more exact measurement using dedicated phantoms [14]. Therefore, quantitative ADC thresholds require validation before applied clinically.

This study compares DWI and DP as diagnostic metrics. Accordingly, we did not investigate lesion morphology. Morphology, however, is essential in the interpretation of lesions in breast MRI. Its ability to distinguish benign from malignant lesions has been established during the last decades, and all relevant morphologic criteria can be assessed on early enhanced images [5, 13, 33]. Future studies should validate our results and may provide an interpretation model integrating IP, morphological assessment, and ADC metrics.

We compared DP and DWI for lesion classification. Beyond this diagnostic setting, our results should be handled with care until validated prospectively. For instance, assessment of DP images may still be required in the assessment of lesion extent and tissue response after neoadjuvant chemotherapy (NAC) [34].

In conclusion, we demonstrated that DWI could substitute DP as a diagnostic metric. Because DWI is typically used as an add-on to IP and DP, our findings have a potential clinical impact. If verified by prospective multireader trials, our results provide a rationale to abbreviate and to simplify current breast MRI practice without losing diagnostic information.

## Abbreviations

ADC: Apparent diffusion coefficient
AUC: Area under the ROC curve
BI-RADS: Breast Imaging Reporting and Data System
CI: 95% confidence interval
CURVE: Combination of IP and DP
CURVE+: Combination of IP, DP, and DWI
DP: Delayed phase
DWI: Diffusion-weighted imaging
EES: Extravascular extracellular space
EPI: Echo planar imaging
EUSOBI: European Society of Breast Imaging
FLASH: Fast low angle shot
FOV: Field of view
Gd: Gadolinium
GRAPPA: Generalized autocalibrating partial parallel acquisition
IP: Initial phase consisting of the precontrast and the first postcontrast scan
IP+: Combination of IP and DWI
MRI: (Breast) magnetic resonance imaging
ROC: Receiver-operating characteristics
ROI: Region of interest
SE: Standard error
SI: Signal intensity
SOR: Standard of reference
TE: Echo time
TR: Repetition time
